# Framework Convention on Tobacco Control 2030—A Program to Accelerate the Implementation of World Health Organization Framework Convention for Tobacco Control in Low- and Middle-Income Countries: A Mixed-Methods Evaluation

**DOI:** 10.1093/ntr/ntad022

**Published:** 2023-02-09

**Authors:** Kamran Siddiqi, Helen Elsey, Mariam A Khokhar, Anna-Marie Marshall, Subhash Pokhrel, Monika Arora, Shirley Crankson, Rashmi Mehra, Paola Morello, Jeff Collin, Geoffrey T Fong

**Affiliations:** Department of Health Sciences, University of York, York, UK; Hull York Medical School, York, UK; Department of Health Sciences, University of York, York, UK; Hull York Medical School, York, UK; Department of Health Sciences, University of York, York, UK; Department of Health Sciences, University of York, York, UK; Brunel University London, Uxbridge, UK; Public Health Foundation India, Delhi, India; Brunel University London, Uxbridge, UK; Brunel University London, Uxbridge, UK; Institute for Clinical Effectiveness and Health Policy, Buenos Aires, Argentina; University of Edinburgh, Edinburgh, UK; University of Waterloo, Waterloo, Canada

## Abstract

**Background:**

Framework Convention on Tobacco Control (FCTC) 2030 Program (2017–2021) was launched to accelerate World Health Organization (WHO) FCTC implementation in 15 low- and middle-income countries (LMICs). We evaluated the Program in six domains: Governance; Smoke-Free Policies; Taxation; Packaging and Health Warnings; Tobacco Advertising, Promotion, and Sponsorship (TAPS) bans; and International and Regional Cooperation.

**Aims and Methods:**

Following a mixed-methods design, we surveyed (June–September 2020) FCTC focal persons in 14 of the 15 countries, to understand the Program’s financial and technical inputs and progress made in each of the six domains. The data were coded in terms of inputs (financial = 1, technical = 1, or both = 2) and progress (none = 1, some = 2, partial = 3, or strong = 4) and a correlation was computed between the inputs and progress scores for each domain. We conducted semi-structured interviews with key stakeholders in five countries. We triangulated between the survey and interview findings.

**Results:**

FCTC 2030 offered substantial financial and technical inputs, responsive to country needs, across all six domains. There was a high positive correlation between technical inputs and progress in five of the six domains, ranging from *r* = 0.61 for taxation (*p* < .05) to *r* = 0.91 and for smoke-free policies (*p* < .001). The interviews indicated that the Program provided timely and relevant evidence and created opportunities for influencing tobacco control debates.

**Conclusions:**

The FCTC 2030 Program might have led to variable, but significant progress in advancing FCTC implementation in the 15 countries. As expected, much of the progress was in augmenting existing structures and resources for FCTC implementation. The resulting advances are likely to lead to further progress in FCTC policy implementation.

**Implications:**

What this study adds: In many LMICs, WHO FCTC policies are not in place; and even when enshrined in law, they are poorly enforced. It is not clear how financial and technical assistance to high tobacco-burden LMICs can most effectively accelerate the implementation of WHO FCTC policies and offer value for money. Bespoke and responsive assistance, both financial and technical, to LMICs aimed at accelerating the implementation of WHO FCTC policies are likely to lead to progress in tobacco control.

## Introduction

Tobacco use damages human health leads to economic losses and causes environmental degradation.^1^ Among more than 1 billion tobacco users in the world, approximately 80% now live in low- and middle-income countries (LMICs).^2^ Although tobacco use has declined in most high-income countries, the number of tobacco consumers is rising in many LMICs.^3^

The WHO Framework Convention on Tobacco Control (FCTC) seeks to reduce the burden of tobacco use through key supply and demand measures laid out in its articles. Key demand measures require governments to raise taxes on tobacco, ban smoking in public, enforce warning labels on tobacco packs, ban tobacco advertising, and offer help in quitting tobacco.^4^ When effectively implemented, several of these measures are strongly associated with reductions in smoking prevalence.^5^ These measures can reduce the global tobacco-related disease burden substantially, and contribute toward advancing the United Nations Sustainable Development Goals (call for action by all countries—poor, rich, and middle income—to promote prosperity and address a range of social needs including education, health, social protection, and job opportunities, while tackling climate change and environmental protection).^6^

Despite being parties to the WHO FCTC, only a few countries have fully implemented the FCTC demand measures.^4,7^ For example, only 34% of countries, covering 24% of the world’s population, have implemented comprehensive smoke-free laws; and only 20% of countries, covering 13% of the world’s population, have tobacco taxes at significantly high levels to have achieved the highest-level of implementation, according to the WHO.^8^ Policies to implement these measures are not in place in many countries; and even when enacted in law, are poorly enforced and implemented.^7,9^

The reasons for this significant “implementation gap” in tobacco control in LMICs are well documented and include tobacco industry’s (TI) constant interference to prevent^10^ or slow^11^ policy development, and a lack of state capacity in tackling this interference and enforcing FCTC policies.^12,13^ On the other hand, there is some evidence that collaboration between state actors and tobacco control advocates can help in protecting policymaking from TI litigations,^14,15^ and build domestic capacity in implementing such policies.^16^ Most of this evidence is based on examples from individual countries pursuing single policies; there is very little evidence of any concerted multicountry efforts to accelerate the implementation of FCTC demand articles. Such evidence is key to mobilizing international financial and technical support in maximizing efforts to implement FCTC policies and realize their full potential worldwide.

The FCTC 2030 Program constitutes a concerted international effort to accelerate the implementation of WHO FCTC articles within 15 LMICs in phase I (Cabo Verde, Cambodia, Chad, Colombia, Egypt, El Salvador, Georgia, Jordan, Madagascar, Myanmar, Nepal, Samoa, Sierra Leone, Sri Lanka, and Zambia) and in nine additional countries in phase II. The Program was designed to build capacity in tobacco control in these countries and offer a suite of supporting materials, tools, and activities to other LMICs facing the tobacco challenge. The FCTC 2030 has received a substantial grant of £15m over 5 years (2016–2021) from the UK government. The program aimed to: Strengthen tobacco control governance, primarily through the implementation of FCTC Article 5 (increase tobacco taxation); implement the two time-bound FCTC measures on tobacco packaging and on ending tobacco advertising, promotion, and sponsorship (TAPS); and support the implementation of other WHO FCTC articles that receive national priority. The program also aimed to strengthen tobacco control efforts, build capacity, secure support for stronger tobacco control legislation, and enhance implementation of the national tobacco control plans in phase 1 countries. This is being done by providing funding, technical assistance, materials, workshops, toolkits, and other forms of assistance. We assessed the implementation and impact of the FCTC 2030 program in six domains: Governance; Smoke-Free Policies; Taxation; Packaging and Health Warnings; TAPS bans; and International and Regional Cooperation.

## Methods

We used a mixed-methods design using both sequential and parallel features,^17^ which was adapted in part from the evaluation framework used by the FCTC Impact Assessment Expert Group.^9^ This included an online questionnaire-based survey of the FCTC focal persons appointed by the respective governments in all phase 1 countries and semi-structured interviews conducted with key stakeholders in five of these countries.

The survey focused on the financial and technical inputs provided by the program and any resulting progress. In addition to covering each of the six key domains, the survey addressed capacity building and TI interference. Following a pilot and after receiving informed consent, FCTC focal persons in 14 of the 15 countries were recruited for the survey. We facilitated and administered the online survey between June 2020 and September 2020. The FCTC focal persons were responsible for completing the survey—one per country. However, to allow for comprehensive and triangulated responses, the focal persons were requested to consult with other potential informants in their respective countries. To allow them time to prepare and consult with others, the questionnaires were sent to the focal persons in advance along with the participant information sheet. Questionnaire completion was facilitated by the research team during a Zoom call. However, if this was not feasible, the focal persons had the option to complete the questionnaires in their own time. Where necessary, all verbal and written communication was carried out in English, French, Portuguese, and Spanish. The data were then translated into English for analysis.

The survey responses were coded by two team members using thematic analysis^18^ and summarized according to the program inputs and the progress made in each country against each of the six domains and their subdomains. For each of the subdomains identified under the six domains, the research team gave a numerical code based on the summarized narratives, as follows: For inputs, countries receiving either financial or technical inputs (e.g. guidance, advice, training, and workshops) were coded as 1 and those receiving both as 2. For progress, those that made no progress were coded as 1, some progress (limited to advocacy, awareness raising, and training) were coded as 2, and those with partial progress (what is coded as 2 *plus* drafting plans, policies, and legislations) were coded as 3 and those with strong progress (what is coded as 2 and 3 *plus* implementing plans, enforcing policies, and changing legislations) were coded as 4. The codes were allocated by two team members independently and any discrepancies were resolved by a third team member. Two heatmaps were created, one for the inputs and one for the progress, depicting these numerical codes for each country. For inputs, the numerical codes given to each of the subdomains were added together to produce a single score for each of the six domains for each country. For progress, the numerical codes given to each of the subdomains were used to produce a mean score for each of the six domains for each country. These input and progress scores for each country were used to estimate a Pearson correlation coefficient for all six domains.

In the selection of five case study countries, we took three criteria into consideration: (1) representation across key WHO regions; (2) capturing the full spectrum of engagement with the FCTC 2030 program (from high to low), based on WHO’s internal assessment, and (3) ability to recruit key in-country stakeholders for online interviews. Based on these criteria, five of the 15 phase 1 countries were selected from four WHO regions (Sierra Leone and Zambia [AFRO], Colombia [PAHO], Jordan [EMRO], and Nepal [SEARO]). Initially, the evaluation team had planned to collect qualitative data through face-to-face interviews during visits to the five selected countries. However, qualitative data collection (October 2020 and April 2021) coincided with the coronavirus disease 2019 pandemic, so interviews were conducted using online video conferencing (Zoom). Interviews were conducted in English in Zambia, Sierra Leone, Jordan, and Nepal and in French, Portuguese, and Spanish in Colombia. These interviews were later transcribed and translated into English.

In line with our mixed-methods design,^17^ triangulation between methods and data sources happened at multiple points within the evaluation. Initially, we were able to utilize a sequential approach where the results of the questionnaire informed the development of the interview guide. The guide covered (but was not limited to): Interactions with the FCTC 2030 program; the impact of the program on tobacco control governance and on tobacco control policies in the country; perceptions of the program (including opinions about the program and what has been the most and least helpful); barriers and challenges to implementation; evaluation of existing measures and monitoring (including how to improve and strengthen measures); and the overall achievements of the program. We set up online meetings with potential interviewees, including parliamentarians, representatives from government ministries, advocacy groups, Civil Society Organisations (CSOs), and academia. In each country, seven to eight interviews were conducted (see Table 1).

**Table 1.** Qualitative Interview Participants

Primary roles	Jordan	Colombia	Zambia	Nepal	Sierra Leone
Ministry of Health (previous and current)	1		2	3	2
Other government ministries, police force		2	2		1
CSOs/NGOs/advocacy group members and tobacco control lawyers	4	3	2		1
Academics	1	2	1	2	2
Members of Parliament/Senator		1	1		1
Medical/public health professionals (inc. WHO)	2			2	
Total participants	8	8	8	7	7

Interviews were transcribed verbatim and analyzed using thematic analysis.^18^ We used a parallel mixed-methods approach where we compared and contrasted qualitative findings with the questionnaire results. This allowed us to check interview transcripts to corroborate the information provided in the survey. We compared our quantitative findings with the emergent themes from the qualitative analysis seeking explanatory or contradictory evidence.

Ethical approval was granted by the University of York Research Governance Committee and from the local ethical committees in each of the five case study countries.

## Results

Of 15 countries where the FCTC 2030 program was implemented, the focal persons in 13 countries completed the questionnaire; one (Madagascar) did not consent to participate and one (Cambodia) completed the taxation section only. Of 13 completing the survey, the focal persons in 10 countries attended the online Zoom meetings while the others completed the questionnaire in their own time. The summarized narratives based on the survey responses from 14 countries are presented in Supplementary Tables 1–6.

Table 2 provides a sum of all inputs; a relatively high score in a domain indicates that the respective country received substantial financial and/or technical input across its several subdomains and vice versa. Table 3 provides mean scores for the progress made for each country under each of the six domains.

**Table 2.** Country-Wise Total Scores Combining Grades for Both technical and Financial *Inputs*

	Governance	Smoke-Free policies	Taxation	Packaging and Health Warnings	TAPS	International and Regional Cooperation
Zambia	6	2	3	0	0	3
Sierra Leone	5	2	3	0	0	3
Jordan	8	3	3	2	2	3
El Salvador	7	3	2	2	2	2
Colombia	5	1	2	2	1	4
Egypt	8	1	1	0	1	2
Myanmar	6	3	3	2	2	3
Samoa	7	4	3	2	3	3
Georgia	8	6	4	3	4	5

TAPS = Tobacco Advertising, Promotion, and Sponsorship.

**Table 3.** Country-Wise Mean Scores for *Progress*

	Governance	Smoke-free policies	Taxation	Packaging and health warnings	TAPS	International and regional cooperation
Zambia	2.8	1.75	3.33	1	1	2.67
Sierra Leone	2.6	1.75	3	1	1	3.33
Jordan	3	2.25	1.33	1	2.33	2
El Salvador	3	2	1.33	3	2	2.67
Colombia	3	1.5	1.33	2	1.33	3.67
Egypt	3.4	1.5	1.33	1	1.33	2.67
Myanmar	2.6	2.5	2.67	2.5	1.33	2.33
Samoa	2.8	3	2.67	2.5	2.67	2.67
Georgia	3	3	1.67	3	3	3.33

TAPS = Tobacco Advertising, Promotion, and Sponsorship.

For each domain, Table 4 provides overall mean scores for technical, financial, and a combination of technical and financial inputs; it also provides correlation coefficients between the two types of inputs. The sum of inputs for Governance was the highest followed by International and Regional Cooperation and Smoke-Free Policies. We also did not find any strong correlation between financial and technical inputs, which indicates that the two types of inputs were distinct and separate sources of support. This reflects the flexible and responsive nature of the inputs offered.

**Table 4.** Overall Mean Scores for Inputs and Progress

Domains	Average of financial inputs	Average of technical inputs	Sum of financial and technical inputs	Correlation of financial inputs and technical inputs	Average of progress
Governance	2.67	3.83	6.5	-0.14	2.98
Smoke-free policies	0.75	2	2.75	-0.26	2.15
Taxation	0.08	2.33	2.41	0.21	2.19
Packaging and health warnings	0.33	1.17	1.5	0.26	2
TAPS bans	0.33	1.17	1.5	0.17	1.69
International/regional cooperation	1.17	1.58	2.85	-0.14	2.69

TAPS = Tobacco Advertising, Promotion, and Sponsorship.

For each domain, Table 5 presents the correlations between the technical and financial inputs and the progress. For all six domains (Governance, Smoke-Free Policies, Taxation, Packaging and Health Warnings, TAPS bans, International and Regional Cooperation), there was a positive correlation between FCTC 2030 inputs and the progress made. Compared to financial inputs, technical inputs were strongly related to progress. In the majority of the domains (five of the six for technical inputs, four of the six for combining financial and technical inputs), this positive correlation was statistically significant despite the low number of countries. The combination of the two types of inputs gave slightly higher correlations with progress. For three of the six domains, the correlation between financial + technical inputs and progress was extremely high (0.94 for TAPS bans, 0.93 for Smoke-Free Policies, and 0.84 for Packaging and Health Warnings), all significant at *p* < .001.

**Table 5.** Pearson Correlation Coefficients Between the Inputs and Progress

	Pearson correlations between inputs and progress		
Domains	Financial inputs and progress	Technical inputs and progress	Financial + technical inputs and progress
Governance	0.36	0.33	0.52^†^
Smoke-free policies	0.04	0.90***	0.93***
Taxation	-0.18	0.61*	0.50^†^
Packaging and health warnings	0.36	0.85***	0.84***
TAPS bans	0.55^†^	0.85***	0.94***
International/regional cooperation	0.34	0.64*	0.73**

^†^ *p* < .10; * *p* < .05; **p < .01; ***p < .001.

TAPS = Tobacco Advertising, Promotion, and Sponsorship.

The interviews provided rich information not only on the program but also on the wider policy environment. The following themes emerged from the parallel analysis of the survey and the interviews providing insights into the implementation and impact of FCTC 2030.

### Strengthening Governance and Establishing National Coordinating Mechanism

The CSOs, government officials, academics, and parliamentarians perceived consistently that the program strengthened cross-government working, making incremental progress in all case study countries. Governance received higher levels of input than other domains but faced significant barriers to making progress (Table 5). The survey suggests that NCMs were established and operationalized in four countries but were partially functioning or nonfunctioning in 10 countries.

I have to say there is no regular coordinating meeting. I don’t know about previously, but since two and a half years...We can blame it now on COVID, but even if there was no COVID, it is not functioning fully *(Medical professional, Nepal).*

Interviewees identified multiple barriers including TI interference, frequent ministerial changes, lack of political will, weak governance structures, and in Nepal, challenges in establishing such structures during major constitutional changes. While progress was slow, it appeared that without FCTC 2030 inputs, in some countries, it could well have been nonexistent.

This would not have happened without FCTC 2030 involvement because the NCM was not active until they were involved. They empowered the committee. *(Public health professional, Jordan).*

### Strengthening the Role of Nongovernment Actors

Engaging nongovernment actors was a key strategy in FCTC 2030. This included drawing on their campaigning skills and networks to expose TI interference (Zambia and Egypt), monitoring tobacco sales (Sri Lanka), raising public awareness of antitobacco laws (Jordan) and tobacco-related harms (Sierra Leone, El Salvador, Samoa, Cabo Verde, and Zambia). Several activities highlighted a clear understanding of the role of CSOs in leveraging action within the government. For example, in Zambia, where CSOs were particularly influential, FCTC 2030 was able to leverage CSO influence to meet senior officials and initiate discussions on policies and tobacco taxation.

What FCTC has done is to clearly indicate the need for participation of various stakeholders not only from government line ministers but also from society. So that has allowed us actually to even invite to our meetings that we have other stakeholders from CSOs, from other international agencies. . . . So the FCTC has just really opened us up to allow the participation of various stakeholders. (*Ministry of Health, Zambia).*

The interactions with CSOs were not always fruitful, particularly where only one prominent CSO was involved (Colombia). There was a lack of integration between CSO activity and government processes in Nepal. Likewise, some meetings with CSOs were not always felt to be productive in Jordan.

FCTC 2030’s role in creating space for local academics to present tobacco-related research to government and nongovernment actors was highlighted in several countries. This was particularly useful in Jordan and Colombia where FCTC was sometimes seen as an external imposition. FCTC 2030 created space for academics to showcase nationally relevant findings grounded in the country context.

What I value the most is that she [FCTC focal point] would create the spaces so I could communicate the results of the research we were doing. . . . She has invited me to participate in the technical committee for the creation, design, and selection of 2021–2022 warning labels. For us, this was what we had always wanted to accomplish: being able to be within the group that, in a way, takes that kind of decisions” *(Academic, Colombia).*

### Facilitating Regional and International Cooperation

FCTC 2030 supported a number of international cooperation events. Government officials, CSOs, and lawyers participated in these events, with 10 focal persons stating partial or full benefit. These interactions were particularly beneficial when chosen to boost capacity and understanding. Sharing strategies to take forward tobacco taxation, establish legal frameworks (eg, Zambia), and tackle TI interference (eg, Brazil and Colombia) were particularly useful.

For countries facing particular barriers such as extensive industry interference and limited political commitment, international cooperation was particularly helpful in building solidarity, motivating government officials, lawyers, and CSOs, and making them feel that they are part of a global tobacco control movement. This was particularly evident where regional cooperation was required, for example building cooperation in southern Africa to find alternatives to tobacco farming. In Sierra Leone, where tobacco control is in its infancy, regional links were found to be particularly helpful in raising awareness of possible strategies.

Well, when we have international meetings, we meet with different countries, I mean we share various experiences. And we’re able to learn from those experiences. For example, what is Gambia doing for tobacco control in their country. We will not be able to get an opportunity like that if FCTC was not involved *(Ministry of Health, Sierra Leone).*

### Strengthening Technical Capacity and Sharing Evidence

All interviewees highlighted the value of timely and appropriate technical support to build skills and provide policy-relevant evidence at the right time to influence key debates and/or policy developments. A valuable input in Jordan, Colombia, and Zambia was the UNDP (United Nations Development Programme)-supported investment cases. This built understanding of the economic impact of tobacco use and the benefits of its control. These perspectives were clearly new to many government officers and helped in prioritizing tobacco control within the Ministry of Finance in Jordan and supported the case for increased taxation in Colombia.

It is difficult to measure how the FCTC, and specifically the investment case, was able to counter those [opponents to tax increases] but to give you an example . . . one of the initiatives two years ago was brought in in Congress...and 10 Congressmen voted against the initiative and 1 voted in favour of the initiative. The last one was also not approved two or three months ago; it was like 6 to 5, and that is progress *(Researcher, Colombia).*

The efforts to strengthen capacity were particularly effective when aligned with specific contextual issues. The studies commissioned on TI interference in Jordan and on alternative livelihoods for tobacco farmers in Zambia were highlighted as catalysts. Using the existing data and sharing it in accessible formats also worked well in Jordan.

The interviewees highlighted that funding stakeholder engagement, training and specialized legal and tax advice were instrumental in making progress in all five countries. The value of the financial inputs was specifically mentioned by government stakeholders; in Sierra Leone and Nepal, the enabling role of FCTC 2030 resources was highlighted.

Inter-sectoral ministerial meetings have helped us talk about tobacco control between different ministries. This includes civil societies and local-level governments. It could have been possible without FCTC 2030, but the fund that the government of Nepal provides to us for Tobacco Control programs every year is quite small. So, it also might not have been possible because of this. *(Ministry of Health and Population, Nepal).*

## Discussion

Our study found that the levels of financial and technical inputs made available by FCTC 2030 were based on the country’s needs and capacity and hence responded to the dynamic nature of tobacco control in the respective settings. Overall, FCTC 2030 support leaned heavily toward strengthening governance, establishing national coordinating mechanisms where necessary, and forging multi-sectoral alliances. Activities specific to a number of WHO FCTC articles ranged from advocacy and awareness-raising campaigns to preparing technical documents and securing political support for policy change. FCTC 2030 played a valuable role in generating, providing, and communicating appropriate evidence to underpin the tobacco control activities and influence of government and non-government actors. Much of FCTC 2030’s technical inputs also helped build capacity through training and targeted communication of evidence to support policy enforcement, tobacco taxation, and legal action. The program facilitated regional and international cooperation specifically in identifying strategies to reduce tobacco farming and addressing TI interference in policymaking. Creating space for academics and civil society to engage in policy dialogue, advocating for change, and organizing tobacco control campaigns were recognized as important contributions from FCTC 2030.

It is widely acknowledged that the ratification of WHO FCTC has led to accelerated implementation of evidence-based tobacco control policies in the past, particularly in enforcing health warning labels on cigarette packs^11^ and banning tobacco advertisements.^12^ It is also recognized that most LMICs, especially those with weak domestic capacity,^13^ require additional support in implementing WHO FCTC. Our study indicates that the technical and financial inputs offered by the program strengthened national infrastructure and capacity to support WHO FCTC implementation in countries that were lagging behind in tobacco control. The program also directed its efforts in building state capacity in dealing with the widely prevalent TI interference in policy making.^19^ Supporting countries in such ways may help to counter allegations that tobacco control represents an “outsider agenda” defined by external actors.^20^

We also found that the activities generated by FCTC 2030 led to substantial changes that progressed the implementation of WHO FCTC in most cases. The achievements included establishing national coordinating mechanisms, securing sector-wide support, policy amendments, tobacco tax increases, and effective implementation of existing policies. For all six domains (Governance, Smoke-free Policies, Taxation, Health Warnings, TAPS ban, and International/Regional Cooperation), there was a positive correlation between FCTC 2030 inputs (especially technical inputs) and the progress made. In the majority of the domains (five of the six for technical inputs, four of the six for combining financial and technical inputs), this positive correlation was statistically significant despite the low number of countries. Our counterfactual approach,^21^ supported the notion that most of these changes would not have happened without the inputs from FCTC 2030. This is in line with the existing albeit scant literature. Among LMICs, Colombia, Nepal, and Uruguay have been able to utilize technical and financial support from international tobacco control agencies^16^ in engaging politicians^14^ and civil society^15^ to mobilize tobacco control efforts and mount an effective defense against TI interference and litigations. Many countries have also worked across different agencies to resolve the inherent tensions between trade and health goals and made significant progress in tobacco control.^22^ The implementation of standardized packaging in Australia was a good example of how the government used a strong interagency coalition to put up a formidable defense against international commercial pressure and litigations against the reform.^23^

We acknowledge that our evaluation has some limitations. The evaluation was commissioned after the start of the program with no opportunity to gather baseline data. In the absence of baseline or comparative data, it is difficult to attribute the progress to program inputs. However, the use of counterfactuals in our survey and interviews^24^ is likely to offer more objectivity to our findings than probabilistic accounts.^25^ Caution must also be exercised when interpreting survey findings, which are largely based on self-reported data received from a single key informant (FCTC focal person) per country. To overcome individual biases, we validated self-report data (where possible) with other documentary evidence. We also asked each focal person to respond to the survey only after consulting with other key informants in the country.

We were unable to measure the potentially substantial impact of the coronavirus disease 2019 pandemic on the achievements of the program. During the pandemic, most governmental resources and attention shifted away from tobacco control and the TI exploited the situation by enhancing its corporate social responsibility activities in many instances.^26^ On the other hand, the pandemic and subsequent lockdowns might have increased people’s interest in quitting tobacco and limited their access to purchasing tobacco and smoking outdoors.^27^ We also acknowledge that the lack of face-to-face qualitative interviews might have limited the depth of information collected. Our use of local researchers within each country to collect data helped to mitigate these limitations. Our evaluation was conducted over a tight time frame, and this meant that we were unable to use a sequential approach when conducting the qualitative and quantitative analysis. This could potentially have provided more depth to some of the quantitative findings, however, the parallel analysis process demonstrated the consistency between the data from the two methods.

We recognize that many countries that need the most inputs often have weak structures, low political and civil society support, and much industry interference at the baseline, which suggests that the real value of the FCTC 2030 investment may not be realized for some time to come.

Our evaluation has some important implications for international donors and public health agencies interested in global tobacco control. Our results indicate that in high tobacco-burden countries with limited capacity and resources, technical and financial assistance are likely to accelerate the implementation of WHO FCTC and should be mobilized. As found in the case of FCTC 2030, any future efforts to support the implementation of WHO FCTC should be based on initial country-level needs assessments that consider the wider political context of tobacco control. A flexible portfolio of inputs that can be adapted to respond to the dynamic tobacco control context is likely to produce the desired effect. Such efforts must also support the generation and targeted dissemination of relevant and locally owned evidence to support tobacco control at national and subnational levels. Strengthening tobacco control governance^28^—the bedrock for tobacco control—must continue to receive priority even if its impacts are less visible in the short term. Future programs directly addressing TI interference in public policies would be well placed to support the effective implementation of WHO FCTC.^19^ To reduce TI interference and promote effective FCTC implementation, countries participating in programs such as FCTC 2030 should adopt a code of conduct or guidelines consistent with Article 5.3 implementation guidance^22^ that are applicable across all government departments.

Our findings indicate that the progress was in general positively and significantly correlated with the level of inputs. Therefore, it can be concluded that the higher the inputs, the more progress a country can make with its tobacco control agenda, provided that the size of the marginal benefits from FCTC 2030 remains justified by the level of inputs received.

## Supplementary Material

A Contributorship Form detailing each author’s specific involvement with this content, as well as any supplementary data, are available online at https://academic.oup.com/ntr.

ntad022_suppl_Supplementary_Tables

## Data Availability

The data belongs to the WHO and cannot be made freely available. Any request for data should be directed to Trinette Lee (WHO FCTC secretariat) at leet@who.int
