# Supplementary material for: Framework Convention on Tobacco Control 2030—A Program to Accelerate the Implementation of World Health Organization Framework Convention for Tobacco Control in Low- and Middle-Income Countries: A Mixed-Methods Evaluation
Source: Nicotine Tob Res. 2023 Feb 9;25(6):1074–81. doi: 10.1093/ntr/ntad022 (PMC10202630; doi:10.1093/ntr/ntad022)
Supplement: ntad022_suppl_Supplementary_Tables [file ntad022_suppl_supplementary_tables.docx]

**Supplemental tables (1-6)**

Tables 1a-e: Governance

| **Table 1a: Governance—National Coordinating Mechanism (NCM)** | | |
| --- | --- | --- |
| **Country** | **Inputs** | **Progress** |
| **Zambia** | Financial and technical support provided to establish the NCM (2) | Accelerated the implementation of tobacco control programmes (3) |
| **Sierra Leone** | Financial and technical support provided to set up a National Multi-sectoral Tobacco Taskforce and appoint a focal person (2) | Under the umbrella of the taskforce, various ministries and sectors came together and developed a tobacco control activity plan (4) |
| **Jordan** | Financial, technical support provided to re-establish NCM (2) | NCM’s terms of reference were developed and a technical committee to oversee the action plan was set up (3) |
| **El Salvador** | Financial and technical support provided to set up the NCM (2) | None observed as the NCM was still in the process of being set up (1) |
| **Colombia** | Technical support provided to strengthen the MoH (1) | Various ministries and economists came together to work on building a professional team (3) |
| **Egypt** | Financial and technical support provided to establish the NCM (2) | NCM’s terms of references were developed for the multi-sectoral coordinating committee to meet regularly (4) |
| **Myanmar** | Needs assessment, financial and technical support provided to re-establish the NCM. (2) | Tobacco control committee was reformed alongside increased parliamentarian engagement (4) |
| **Samoa** | Financial and technical support provided to set up the NCM (2) | None specified (1) |
| **Georgia** | Financial, technical support and toolkits provided; workshops organised (2) | A tobacco control group was established (3) |
| **Sri Lanka** | NATA (National Authority on Tobacco and Alcohol) was already established in 2006 prior to FCTC 2030; thus, no support was required (0) | - (1) |
| **Cabo Verde** | Financial and technical support provided to MoH to set up NCM (2) | A fully functional NCM was established (4) |
| **Chad** | Financial, technical support provided to re-establish NCM (2) | A regulation was drafted to elevate NCM under the office of the Prime Minister (3) |
| **Nepal** | NCM was already established prior to FCTC 2030. Therefore, no support was required (0) | - (1) |
| **Cambodia (taxation only)** |  |  |

| **Table 1b: Governance—National Tobacco Control Strategy/Plan** | | |
| --- | --- | --- |
| **Country** | **Inputs** | **Progress** |
| **Zambia** | Financial support provided to develop a national tobacco control strategic plan (1) | Supply and demand reduction measures were identified as part of the strategic plan (3) |
| **Sierra Leone** | Technical support provided to strengthen the NCD strategic plan (1) | The plan identified priority areas for interventions for tobacco control (3) |
| **Jordan** | Financial and technical support provided to develop a national tobacco control plan (2) | A tobacco control strategy/plan drafted; currently put on hold due to COVID-19 (3) |
| **El Salvador** | Technical support provided to develop a national tobacco control plan (1) | The plan identified priority areas for policy interventions for tobacco control (4) |
| **Colombia** | None specified (0) | - (1) |
| **Egypt** | Financial support provided to accelerate the development of a national tobacco control plan (1) | Tobacco control indicators were identified as part of the plan (3) |
| **Myanmar** | Financial and technical support provided to review the 2000 policy (2) | A new policy is under-development (3) |
| **Samoa** | Financial support for tobacco control policy plan provided to develop tobacco control plan of action (1) | Work began on cessation and implementing FCTC Article 5.3 guidelines (3) |
| **Georgia** | Financial and technical support provided; expert advice and coordination through planning workshops (2) | A tobacco control strategy/plan drafted (3) |
| **Sri Lanka** | Financial support provided to develop a national tobacco control strategy 2020-2025 (1) | The plan identified priority areas of interventions for tobacco control (4) |
| **Cabo Verde** | Financial support provided to develop a national tobacco control plan (1) | The plan developed guidelines and identified priority areas for policy interventions (4) |
| **Chad** | Financial and technical support provided to develop a tobacco control strategy/plan (2018-2022) (2) | The plan identified priority areas for interventions for tobacco control (4) |
| **Nepal** | Financial support provided to develop a multi-sectoral strategic action plan on tobacco control (1) | The action plan was drafted; currently put on hold due to COVID-19 (3) |
| **Cambodia (taxation only)** |  |  |

| **Table 1c: Governance—Multi Sectoral Participation** | | |
| --- | --- | --- |
| **Country** | **Inputs** | **Progress** |
| **Zambia** | Financial support provided to hold coordination meetings; technical advice provided (2) | NCM’s terms of references developed for the coordinating committee to meet regularly (3) |
| **Sierra Leone** | Advice provided on how to bring multiple sectors and ministries together (1) | Continued support for the working of National Multi-sectoral Tobacco Taskforce (2) |
| **Jordan** | Advice provided by UNDP and relevant training and workshops organised (1) | A national committee was established to ensure sustainability of the FCTC 2030 programme (4) |
| **El Salvador** | Technical support provided to strengthen multi-sectoral participation (1) | Increased buy-in observed from the non health sectors and stakeholders such as the Ministry of Finance (MoF), Ministry of Education (MoE) and CSOs (4) |
| **Colombia** | Advice sought from other countries, tobacco control organisations and sectors (1) | Effective communication between sectors external to MoH (4) |
| **Egypt** | Financial and technical support provided (2) | A tobacco control strategy/plan drafted; currently on hold due to COVID-19 (3) |
| **Myanmar** | Advice on need for multi-sectoral approach, technical input and documentation provided (1) | A multi-sectoral committee was operational (4) |
| **Samoa** | Financial support for a coordinator to link across ministries and provide technical advice (2) | Greater access to cabinet and higher levels of governance (4) |
| **Georgia** | Advice provided on strengthening tobacco control policy (1) | Multi-sectoral participation strengthened; progress was made in monitoring tobacco control (4) |
| **Sri Lanka** | Financial support provided to hold expert consultations and strengthen multi-sectoral collaborations (1) | Under the umbrella of multi-sectoral collaborations, the need for a national tobacco control strategy was highlighted (2) |
| **Cabo Verde** | Training provided to the multi-sectoral working group on FCTC Article 5.3. A consultant was hired to support annual tobacco control plans (1) | Increased awareness of the working group about the Tobacco Industry tactics (3) |
| **Chad** | Financial support provided to hold expert consultations and multi-sectoral collaborations (1) | Formation of a multisectoral committee allowed countering the Tobacco Industry interference in policy making (4) |
| **Nepal** | Financial support provided to bring multiple sectors and ministries together (1) | Improved communication and understanding amongst ministries and sectors (3) |
| **Cambodia (taxation only)** |  |  |

| **Table 1d: Governance—Civil society engagement** | | |
| --- | --- | --- |
| **Country** | **Inputs** | **Progress** |
| **Zambia** | Advice given on how to better engage with and strengthen civil society (1) | Civil society continued exposing Tobacco Industry’s interference in policy (4) |
| **Sierra Leone** | Advice on how to better engage with and strengthen civil society provided (1) | Civil society was better able to conduct awareness raising activities (3) |
| **Jordan** | Financial support and advice provided on how to better engage with civil society and strengthen the relationships (2) | Additional collaborations with NGOs (3) |
| **El Salvador** | Advice provided on how to better engage with and strengthen civil society (1) | Civil society was better able to conduct awareness raising activities (3) |
| **Colombia** | Financial support and advice provided on how to better engage with civil society and strengthen the relationships (2) | Civil society increased activism in Congress (parliament) and grabbed media attention for tobacco control efforts (4) |
| **Egypt** | Advice provided to MoH on how to better engage with and strengthen civil society (1) | Civil society ran a social media campaign against Tobacco Industry interference in policy (4) |
| **Myanmar** | Existing engagement with a large NGO, so no further support needed (0) | None specified.(1) |
| **Samoa** | Advice on how to better engage with civil society provided (1) | Civil society is better able to conduct awareness raising activities (3) |
| **Georgia** | Financial and technical support provided; toolkits, materials and coordination through planning workshop (2) | More collaborations with civil society and non governmental partners (3) |
| **Sri Lanka** | Advice given on how to better engage with and strengthen civil society (1) | Civil society conducted field level rapid assessment study on tobacco retail patterns (4) |
| **Cabo Verde** | Advice given on how to better engage with and strengthen civil society (1) | Civil society carried out tobacco control awareness campaigns on social media, television and radio (3) |
| **Chad** | Financial support and advice provided on how to better engage with civil society and strengthen the relationships (2) | Civil society recognised and empowered as an important entity to work on tobacco control (3) |
| **Nepal** | No plans on engaging with civil society organisations. (0) | - (1) |
| **Cambodia (taxation only)** |  |  |

| **Table 1e: Governance—Countering Tobacco Industry interference** | | |
| --- | --- | --- |
| **Country** | **Inputs** | **Progress** |
| **Zambia** | No direct financial or technical support provided (0) | None specified (1) |
| **Sierra Leone** | None specified (0) | - (1) |
| **Jordan** | Advice given on how to better engage with and strengthen CSOs (1) | CSOs continued exposing Tobacco Industry’s interference in public policy (2) |
| **El Salvador** | Financial and technical support provided to monitor and curb Tobacco Industry tactics (2) | A protocol for public servants to counter Tobacco Industry interference was prepared (3) |
| **Colombia** | Policy on FCTC Article 5.3 existed; CSOs was supported in advocacy efforts (1) | CSOs helped in curtailing Tobacco Industry’s interference in public policies (3) |
| **Egypt** | Financial and technical support provided to monitor Tobacco Industry interference (2) | National observatory was established to monitor the Tobacco Industry interference (3) |
| **Myanmar** | Financial support provided to organise workshops and print code of conduct and to monitor Tobacco Industry interference (1) | None specified (1) |
| **Samoa** | Technical support provided to support the government services commission to establish a code of conduct for government officers to refuse donations from the Tobacco Industry (1) | FCTC Article 5.3 guidelines implemented (3) |
| **Georgia** | Technical support provided to counter Tobacco Industry interference (1) | A Government decree for Tobacco Industry interference was drafted (this was not approved) (2) |
| **Sri Lanka** | Financial support provided to develop surveillance and national guidelines (1) | Geographic Information System (GIS) based mobile app was developed for surveillance of Tobacco Industry interference  National guidelines to prevent Tobacco Industry interference in public policies were also established (3) |
| **Cabo Verde** | Workshop on countering Tobacco Industry tactics arranged for the staff at MoH and MoF. (1) | Brochure on Tobacco Industry tactics and ways to counter them formulated and disseminated (3) |
| **Chad** | No support was received in this area as there was already a policy on FCTC Article 5.3.  Helped with raising awareness and advocacy through CSOs involvement (1) | Civil society helped in monitoring and curbing Tobacco Industry’s interference in public policies (3) |
| **Nepal** | None specified (0) | - (1) |
| **Cambodia (taxation only)** |  |  |

Tables 2a-d: Smoke-Free Policies

| **Table 2a: Smoke-Free Policies —Sensitisation** | | |
| --- | --- | --- |
| **Country** | **Inputs** | **Progress** |
| **Zambia** | No support needed as the government carried out sensitisation for smoke-free policy (0) | - (1) |
| **Sierra Leone** | Few workshops on smoke free policy were organised (1) | Increased buy-in from parliamentarians to support a comprehensive smoke free policy (2) |
| **Jordan** | Technical support, resources, training, workshops, and technical/expert advice provided (1) | The MoH developed the regulations for smoke free public places and trained the inspectors (3) |
| **El Salvador** | Financial and technical support provided to municipalities to promote smoke free spaces (2) | Increased buy-in from the municipalities with high population density and tourist impact (3) |
| **Colombia** | Technical support, resources, training, workshops, and technical/expert advice provided (1) | An instrument (checklist of measures) developed by the MoH to evaluate and monitor smoke free areas. (3) |
| **Egypt** | None specified (0) | - (1) |
| **Myanmar** | No support needed as sensitisation for smoke-free policy was already underway (0) | - (1) |
| **Samoa** | Some smoke-free laws in place before FCTC 2030. Support provided to advocate for smoke free places was provided; UNDP investment case (1) | Able to advocate for tobacco control policies in the highest levels of governance through the cabinet (3) |
| **Georgia** | Financial support, resources, materials, training, sensitisation, workshops, and technical/expert advice provided (2) | A good communication campaign started. Printing and distribution of materials and several training sessions were conducted on social media as well (3) |
| **Sri Lanka** | Not a part of the application to the FCTC (0) | - (1) |
| **Cabo Verde** | Financial support provided to carry out awareness raising campaigns (1 | National coverage on TV and radio to promote smoke free spaces in public and workplaces (3) |
| **Chad** | Technical and financial support provided to help protect the environment via ban on smoking in public areas. A consultant was hired to help pass the legislation (1) | Legislation on smoke free areas was passed at the national level (4) |
| **Nepal** | No support needed as sensitisation for smoke-free policy was already being carried out by the government (0) | - (1) |
| **Cambodia (taxation only)** |  |  |

| **Table 2b: Smoke-Free Policies —Policy Development** | | |
| --- | --- | --- |
| **Country** | **Inputs** | **Progress** |
| **Zambia** | Financial and technical support provided to strengthen the existing ban and make it comprehensive (2) | A complete ban on smoking in government, educational and health-care facilities, universities, restaurants, public transports, pubs, and bars was introduced (4) |
| **Sierra Leone** | Technical support provided by the International Legal Consortium to help develop a comprehensive smoke free policy (1) | The support prevented the Tobacco Industry from taking advantage of any loopholes in the legislation (3) |
| **Jordan** | Technical support provided for a stronger smoke free policy (1) | New collaborations with the University of Bath and the Bloomberg Institute to enhance technical capacity (2) |
| **El Salvador** | Technical support provided to strengthen regulations for smoke free spaces (1) | The support identified gaps and proposed reforms in the existing law (3) |
| **Colombia** | No support was required as a smoke free policy existed prior to FCTC 2030 (0) | - (1) |
| **Egypt** | Financial support provided to hire a consultant to review smoke free policy (1) | An implementation strategy was developed (3) |
| **Myanmar** | Technical support, workshops and UNDP investment case (1) | Policy strengthened to increase smoke free areas (4) |
| **Samoa** | Technical support given to draft tobacco control policy that included smoke-free places (1) | Five-year work plan in place and tobacco control policy (including smoke free) drafted (4) |
| **Georgia** | Technical and expert advice provided for a comprehensive smoke free policy (1) | There are fewer loop-holes and thus, reduced vulnerability to Tobacco Industry interference (3) |
| **Sri Lanka** | Not a part of the application to the FCTC. However, financial support was provided to meet technical experts on enforcement of tobacco control laws (1) | Recommendations were provided on improving the current smoke-free laws and their implementation (3) |
| **Cabo Verde** | Technical support provided for a workshop to develop a comprehensive smoke free policy (1) | The educational and public health institutes started to establish smoke free spaces at their premises (3) |
| **Chad** | Technical support provided for a stronger smoke free policy at both national and provincial levels (1) | Work started on implementing smoke free law at the provincial level; currently on hold due to COVID-19 (3) |
| **Nepal** | Plans to strengthen smoke free policy were put in place for the later part of 2020 (0)) | - (1) |
| **Cambodia (taxation only)** |  |  |

| **Table 2c: Smoke-Free Policies —Capacity Strengthening** | | |
| --- | --- | --- |
| **Country** | **Inputs** | **Progress** |
| **Zambia** | None specified (0) | - (1) |
| **Sierra Leone** | None specified (0) | - (1) |
| **Jordan** | Training held for the inspectors to ensure smoke free public areas (1) | Increased capacity strengthening (3) |
| **El Salvador** | None specified (0) | - (1) |
| **Colombia** | None specified (0) | - (1) |
| **Egypt** | None specified (0) | - (1) |
| **Myanmar** | Training held at McCabe for lawyers on smoke-free policies and enforcement mechanisms (1) | MoH officials are more engaged (3) |
| **Samoa** | Training held at McCabe for the FCTC focal person  Advice and support provided to staff to identify different ways to advocate for smoke-free policies (1) | Policy drafted (3) |
| **Georgia** | Training held on using social-media to increase awareness of smoke-free policies.  Training for the Ministries of Internal Affairs and Finance to enforce smoke-free laws (1) | Increased awareness among the ministries (3) |
| **Sri Lanka** | Not a part of the application to the FCTC. However, financial support was provided for a study visit of enforcement officers to Singapore.  Exchanging experiences on enforcement of tobacco control laws was deemed valuable (1) | - (2) |
| **Cabo Verde** | None specified (0) | - (1) |
| **Chad** | Training held for people in charge of the public spaces (1) | Increased capacity strengthening  Increased awareness of the importance of having smoke free public spaces (3) |
| **Nepal** | None specified (0) | - (1) |
| **Cambodia (taxation only)** |  |  |

| **Table 2d: Smoke-Free Policies —Enforcement** | | |
| --- | --- | --- |
| **Country** | **Inputs** | **Progress** |
| **Zambia** | None specified (0) | - (1) |
| **Sierra Leone** | None specified (0) | - (1) |
| **Jordan** | None specified (0) | - (1) |
| **El Salvador** | None specified (0) | - (1) |
| **Colombia** | None specified (0) | - (1) |
| **Egypt** | None specified (0) | - (1) |
| **Myanmar** | Conducted training to develop enforcement mechanisms (1) | Greater awareness (2) |
| **Samoa** | Enforcement officers received training in Fiji on smoke free policy implementation from WHO, not from the FCTC 2030 (1) | Limited as enforcement officers have limited power; recommend training police is needed for enforcement (2) |
| **Georgia** | Financial support and resources provided to engage with international experts  Translation of FCTC guidelines into Georgian (2) | Increased knowledge of how to enforce the law including clarity of definitions following the translation of guidelines (3) |
| **Sri Lanka** | None specified (0) | - (1) |
| **Cabo Verde** | None specified (0) | - (1) |
| **Chad** | None specified (0) | - (1) |
| **Nepal** | A plan to form a committee for stronger policy enforcement in place; currently on hold due to COVID-19 (0) | - (1) |
| **Cambodia (taxation only)** |  |  |

Tables 3a-c: Taxation

| **Table 3a: Taxation —Policy Development** | | |
| --- | --- | --- |
| **Country** | **Inputs** | **Progress** |
| **Zambia** | Training, technical/expert advice provided (1) | The MoF considered a gradual tax increase (3) |
| **Sierra Leone** | Technical, technical/ expert advice provided; advocacy for an increase in tobacco taxation (1) | The MoF started considering an increase in taxation (2) |
| **Jordan** | Meetings and workshops held to revise the tax structure and propose new taxes (1) | None, the government decided not to increase any taxes (1) |
| **El Salvador** | A mission was held to support the proposal of an increase in tobacco taxation (1) | The MoF started to evaluate the possibility of gradual increase in tax (2) |
| **Colombia** | Technical support and findings from the investment case supported tripling of the tax  A few workshops with government stakeholders, including the Ministry of Development (DNP), MoF, National Tax Agency (DIAN), and Customs Police (POLFA) were also held. (1) | None, the tax proposal was rejected by the Congress (2) |
| **Egypt** | Not part of the application to the FCTC. However, the investment case assisted in providing information on the cost effectiveness of taxation policy.(1) | Some progress was made but further discussions were put on hold due to COVID-19.(2) |
| **Myanmar** | Needs assessment with information on price increase and tax measures; sharing evidence-based documents; UNDP investment case on taxation and technical support to develop policy recommendations (1) | Greater political support; triggered the Presidential office letter to the Ministry of Planning, Finance and Industry to develop a taxation plan  The investment case enabled policy makers to see the potential positive impact of tax on the poorest (3) |
| **Samoa** | Technical and expert advice provided; UNDP investment case (1) | Able to use the investment case to advocate for price and tax policy  FCTC 2030 experts were able to bring together finance, commerce and industry actors and found compromises to progress taxation policy.  A new work plan including taxation drafted (3) |
| **Georgia** | Administrative & financial documents and support provided  Financial support resources, materials, training, technical/expert advice provided. Translation of FCTC guidelines into Georgian (2) | The Focal person and MoH were able to respond to the challenges to increased taxation raised by the Tobacco Industry (2) |
| **Sri Lanka** | Not a part of the application to the FCTC (0) | - (1) |
| **Cabo Verde** | Technical, technical/ expert advice provided to the MoF; investment case conducted (1) | The MoF started considering an increase in taxation  The investment case allowed gathering of relevant and evidence-based information on increasing taxation (3) |
| **Chad** | The Knowledge Hub carried an in-country technical support mission on tobacco taxation (1) | The government announced a specific excise tax of 100 Francs on all cigarette packs and earmarked the additional revenue for the promotion of public health (4) |
| **Nepal** | Work on taxation policy currently on hold due to COVID-19 (0) | - (1) |
| **Cambodia (taxation only)** | Financial support provided to organise workshops on taxation (1) | Supervision of tax stamps started in various provinces (3) |

| **Table 3b: Taxation —Implementation** | | |
| --- | --- | --- |
| **Country** | **Inputs** | **Progress** |
| **Zambia** | Insights on taxation from the investment case on taxation provided (1) | Tobacco tax on cigarettes went up from 37% in 2016 to 41% in 2018 (4) |
| **Sierra Leone** | Advice provided on building and increasing public and political support (1) | Tobacco excise tax increased by 30% in 2018 (4) |
| **Jordan** | Investment case assisted in building political and public support (1) | More political support and willingness to increase tax, but still the tax was not increased (2) |
| **El Salvador** | The reform of the taxation law was not presented to the legislative Congress (0) | - (1) |
| **Colombia** | Colombia did not implement a tax increase during FCTC 2030 (0) | - (1) |
| **Egypt** | - (0) | - (1) |
| **Myanmar** | Insights on taxation from the investment case on taxation provided  Needs assessment provided information on price increase and tax measures (1) | Some taxes increased. (4) |
| **Samoa** | Advice provided on building and increasing public and political support (1) | MoF and the Ministry of Commerce, Trade and Industry came together on a common mandate of tax increase  A licence fee has been introduced which has been paid by one company (BAT) and inability to pay may force others (a Chinese company) to pull out of Samoa (4) |
| **Georgia** | Advice provided to the Minister of Finance on taxation (1) | Increase in tax underway  Higher prices of tobacco products  FCTC 2030 support has been valuable, however government plans to join the EU have also supported tobacco taxation implementation (2) |
| **Sri Lanka** | - (0) | - (1) |
| **Cabo Verde** | Advice provided on building and increasing public and political support (1) | Ad-Valorem tax increased from 20% to 30% in 2017; and from 30% to 50% in 2019 (4) |
| **Chad** | The WHO Knowledge Hub provided expert advice to various stakeholders (1) | Regular tax was raised from 24% of the retail price in 2018 to 50% in 2019 (4) |
| **Nepal** | - (0) | - (1) |
| **Cambodia (taxation only)** | None specified |  |

| **Table 3c: Taxation —Capacity Strengthening** | | |
| --- | --- | --- |
| **Country** | **Inputs** | **Progress** |
| **Zambia** | Training provided in Cape Town on taxation (1) | The training helped with capacity building for taxation (3) |
| **Sierra Leone** | Training provided in Cape Town on taxation (1) | The training helped with capacity building for taxation (3) |
| **Jordan** | Training of MoF on taxation provided (1) | No change, the King did not allow any tax increase (1) |
| **El Salvador** | Various stakeholders and multi disciplinary teams from government agencies came together to analyse an increase in tax (1) | None, as the proposal was not presented to the legislative congress (1) |
| **Colombia** | A training session was planned for the end of 2020 to enhance technical capacity for the taxation policy (1) | - (1) |
| **Egypt** | - (0) | - (1) |
| **Myanmar** | Training provided in Cape Town on taxation (1) | None specified (1) |
| **Samoa** | Arranged for MoH and MoF staff to attend a taxation workshop in South Africa- cancelled due to COVID travel restrictions (1) | - (1) |
| **Georgia** | Training of MoF on taxation provided (1) | None specified (1) |
| **Sri Lanka** | - (0) | - (1) |
| **Cabo Verde** | Training of MoF and MoH officials in Cape Town on taxation v | A technical report with recommendations for a progressive increase in taxation was formulated and distributed to the relevant government ministries (4) |
| **Chad** | None specified (0) | - (1) |
| **Nepal** | - (0) | - (1) |
| **Cambodia (taxation only)** | None specified (0) | - (1) |

Tables 4a-b:Packaging and Health warnings

| **Table 4a: Packaging and heath warnings —Design and Development** | | |
| --- | --- | --- |
| **Country** | **Inputs** | **Progress** |
| **Zambia** | Not a part of the application to the FCTC (0) | - (1) |
| **Sierra Leone** | Not a part of the application to the FCTC (0) | - (1) |
| **Jordan** | Technical support and documents provided alongside financial support for workshops held at the Dead Sea and Cairo for pictorial health warnings (2) | None, as the Tobacco Industry interfered (1) |
| **El Salvador** | Technical support provided alongside documents for pictorial health warnings (1) | Health warnings were selected on the basis of scientific evidence (3) |
| **Colombia** | Technical support and documents provided for plain packaging and pictorial health warnings (1) | More political willingness to increase the size of the existing 30% coverage of pictorial health warnings (2) |
| **Egypt** | Not a part of the application to the FCTC (0) | - (1) |
| **Myanmar** | Regional meetings in Thailand; FCTC Secretariat support to draft plain pack notification; Myanmar images were used (1) | Recommendations were made to introduce plain packaging by the Union Minister (3) |
| **Samoa** | Resources and technical support provided for plain packaging (1) | While there has been no action yet, plain packaging is within the work plan (2) |
| **Georgia** | Financial support/resources, materials and expert/technical support for pictorial health warnings provided, including focus groups to identify most appropriate pictorial warnings (2) | Enhanced technical capacity for health warning policy  Georgia country-specific and effective pictorial warnings formulated (3) |
| **Sri Lanka** | Technical and financial support to organise training on developing a plain packaging policy (2) | Training helped draft legislation on plain packaging according to the FCTC guidelines (3) |
| **Cabo Verde** | Due to pending approval of the legislation, no relevant activity started by the time of survey completion  Plans were in place for the later part of 2020 (0) | - (1) |
| **Chad** | Technical support and documents alongside financial support provided to hold meetings and discussions (2) | A new order was issued by the MoH to rotate two new pictorial warnings.(4) |
| **Nepal** | No support needed  The work on developing and strengthening policy was carried out by the government prior to FCTC 2030. (0) | - (1) |
| **Cambodia (taxation only)** |  |  |

| **Table 4b: Packaging and health warnings —Building Political Support** | | |
| --- | --- | --- |
| **Country** | **Inputs** | **Progress** |
| **Zambia** | Not a part of the application to the FCTC (0) | - (1) |
| **Sierra Leone** | Not a part of the application to the FCTC (0) | - (1) |
| **Jordan** | Because of the Tobacco Industry interference, no further activity in these areas took place to implement a stronger policy (0) | - (1) |
| **El Salvador** | Advice provided to support a pictorial health warning covering 50% of the cigarette pack (1) | The health warning impacted consumers' decision to buy cigarettes (3) |
| **Colombia** | Technical advice provided to work on increasing the size of health warnings (1) | Growing political support to increase health warnings from 30% to 70%, however this did not succeed in becoming a legislation (2) |
| **Egypt** | Not a part of the application to the FCTC (0) | - (1) |
| **Myanmar** | Fact-sharing meetings between the FCTC team and the Union minister were held (1) | None specified (2) |
| **Samoa** | Expert advice provided to the focal person to make a presentation on health warnings in front of the MoF and the cabinet office (1) | Increased political support from the cabinet; however changes of key office holders (e.g.the Speaker of the House) undermined progress. (3) |
| **Georgia** | Being part of FCTC 2030 helped raise the profile of Georgia’s work on tobacco control and the international support added weight to their activities (1) | Able to advocate at high levels within the government, including the Prime Minister (3) |
| **Sri Lanka** | Because of the Tobacco Industry interference, the implementation was delayed for up to three years(0) | - (1) |
| **Cabo Verde** | - (0) | - (1) |
| **Chad** | Legislation already existed, but FCTC 2030 helped in implementation of stronger pictorial warnings (1) | Considerable progress was made due to the implementation of stronger pictorial warnings (4) |
| **Nepal** | - (0) | - (1) |
| **Cambodia (taxation only)** | - | - |

Tables 5a-c: TAPS ban

| **Table 5a: TAPS ban—Strengthening Policy** | | |
| --- | --- | --- |
| **Country** | **Inputs** | **Progress** |
| **Zambia** | Not a part of the application to the FCTC (0) | - (1) |
| **Sierra Leone** | Not a part of the application to the FCTC (0) | - (1) |
| **Jordan** | Technical support provided for ban on TAPS (1) | The MoH drafted the law to ban tobacco sponsorship; this was not part of the existing laws (3) |
| **El Salvador** | Technical support provided for ban on TAPS (1) | Many ministries and sectors came together to support a TAPS ban. A law reform was drafted (3) |
| **Colombia** | Technical support, advice and training provided for strengthening the ban on TAPS. (1) | Improved communication with local authorities to promote a stronger ban on TAPS (2) |
| **Egypt** | Technical support provided to develop national TAPS guidelines (1) | A plan to disseminate the guidelines was developed; currently on hold due to COVID-19 (2) |
| **Myanmar** | Previous work on development of TAPS policy, TAPS not included in the application, so FCTC 2030 helped on implementation (0) | - (1) |
| **Samoa** | A policy already in place since 2008, so FCTC 2030 provided resources and technical support to strengthen the policy for a ban on TAPS (1) | Some progress was made in strengthening TAPS ban (2) |
| **Georgia** | Financial support/resources, materials and expert/technical support given for TAPS ban (2) | Translation of TAPS materials and dissemination on social media (3) |
| **Sri Lanka** | Financial and technical support alongside expert advice given to strengthen TAPS ban (2) | A technical mission provided recommendations to improve the enforcement of the TAPS ban (3) |
| **Cabo Verde** | Due to pending approval of the legislation, no relevant activity had started by the time of survey completion. Plans were in place for the later part of 2020 (0) | - (1) |
| **Chad** | Due to pending approval of the legislation, no relevant activity had started by the time of survey completion (0) | - (1) |
| **Nepal** | No support needed. The work on developing and strengthening policy was carried out by the government prior to FCTC 2030 (0) | - (1) |
| **Cambodia (taxation only)** | - | - |

| **Table 5b: TAPS ban—Building Political Support** | | |
| --- | --- | --- |
| **Country** | **Inputs** | **Progress** |
| **Zambia** | - (0) | - (1) |
| **Sierra Leone** | - (0) | - (1) |
| **Jordan** | Technical support provided (1) | Increased political support; the Prime Minister issued a circular in support of the ban on TAPS (3) |
| **El Salvador** | Technical support provided to organise meetings (1) | Increased political support. High level meetings were held with government officials to promote a ban on TAPS (2) |
| **Colombia** | None specified (0 | - (1) |
| **Egypt** | Meetings and discussions on hold due to COVID -19 (0) | A plan to disseminate the guidelines was developed; currently on hold due to COVID-19 (1) |
| **Myanmar** | Guidelines on TAPS; workshop on smokeless tobacco and TAPS ban in India (1) | Increased awareness among parliamentarians (2) |
| **Samoa** | Expert advice on health education provided (1) | Built public support (2) |
| **Georgia** | - (0) | During the time of the programme tobacco advertising, posters on the street, tobacco adverts outside of shops and sponsorship reduced (3) |
| **Sri Lanka** | Technical support and expert advice provided (1) | Increased political support- discussions on strengthening a ban on TAPS started taking place at various ministries (3) |
| **Cabo Verde** | - (0) | - (1) |
| **Chad** | - (0) | - (1) |
| **Nepal** | - (0) | - (1) |
| **Cambodia (taxation only)** | - | - |

| **Table 5c: TAPS ban—Implementation** | | |
| --- | --- | --- |
| **Country** | **Inputs** | **Progress** |
| **Zambia** | - (0) | - (1) |
| **Sierra Leone** | - (0) | - (1) |
| **Jordan** | None specified (0) | - (1) |
| **El Salvador** | None specified (0) | - (1) |
| **Colombia** | None specified (0) | - (1) |
| **Egypt** | Meetings and discussions currently on hold due to COVID -19 (0) | - (1) |
| **Myanmar** | Technical support provided to strengthen TAPS ban (1) | None specified (1) |
| **Samoa** | Resources provided (1) | Tobacco Industry stopped advertising at sporting events and focal person warned organisations allowing TAPS (4) |
| **Georgia** | Financial support, resources, materials, training, sensitization, workshops, and technical/expert advice provided (2) | Stronger TAPS ban policy underway (3) |
| **Sri Lanka** | None specified (0) | - (1) |
| **Cabo Verde** | - (0) | - (1) |
| **Chad** | - (0) | - (1) |
| **Nepal** | - (0) | - (1) |
| **Cambodia (taxation only)** |  |  |

Tables 6a-c: International & Regional Cooperation

| **Table 6a: International and Regional Cooperation—International and Regional Meetings & Liaisons** | | |
| --- | --- | --- |
| **Country** | **Inputs** | **Progress** |
| **Zambia** | Financial support provided to attend training at McCabe Institute (Australia), regional (India) and international (Cape Town) workshops and link with Campaign For Tobacco Free Kids (CTFK) (20 | Capacity strengthening- improved participation in the international workshop on taxation (4) |
| **Sierra Leone** | Introduced to the International Legal Consortium (1) | Improved international relations and collaborations (4) |
| **Jordan** | Financial support/resources, materials, expert/technical support provided. Expert and technical support provided to attend a mission in Turkey on taxation (2) | Increased access to officials in other countries like Egypt, Turkey, Colombia, Brazil and Georgia on matters of smoke free policy, taxation, health warnings and Tobacco Industry interference (4) |
| **El Salvador** | Introduced to the teams in Brazil and Colombia to discuss tobacco control issues such as taxation. (1) | Improved international relations and collaborations (4) |
| **Colombia** | Administrative & financial documents and support provided. Financial support, resources, materials, training, technical/expert advice provided (2) | The focal person and MoH feel able to respond to the challenges to increased taxation raised by the Tobacco Industry (4) |
| **Egypt** | Advice provided to link with UNDP and SouthEast Asia Tobacco Control Alliance (SEATCA) to develop an observatory for Tobacco Industry interference as mentioned above (1) | SEATCA’s Tobacco Industry Interference (TII) scorecard was used to review Tobacco Industry’s interference in policy (4) |
| **Myanmar** | Financial support provided to attend regional (India) and international (Cape Town) workshops and link with SEATCA (1) | Improved participation in the regional and international meetings on plain packaging (3) |
| **Samoa** | A meeting of parties for the Pacific Islands was held. Samoa was the only Pacific Island signed up to FCTC, so their role was advocacy within the region (1) | Some progress was made through advocacy (2) |
| **Georgia** | Financial support/resources, materials, expert/technical support provided. Expert and technical support provided for workshops to host various countries in Georgia (2) | Strengthened cooperation and information sharing with other countries. Use of Canadian and New Zealand health warnings in packaging (4) |
| **Sri Lanka** | Financial support provided for two lawyers to attend training at the McCabe Institute (Australia) (1) | Capacity strengthening- improved participation in the international workshop on taxation (3) |
| **Cabo Verde** | Financial support provided to attend workshops in Uganda, Thailand, El Salvador and Georgia (1) | Improved international relations and collaborations (4) |
| **Chad** | None specified (0) | - (1) |
| **Nepal** | None specified (0) | - (1) |
| **Cambodia (taxation only)** |  |  |

| **Table 6b: International and Regional Cooperation—Knowledge Exchange Activities** | | |
| --- | --- | --- |
| **Country** | **Inputs** | **Progress** |
| **Zambia** | None specified (0) | - (1) |
| **Sierra Leone** | Financial support provided to attend workshops in Cape Town (1) | Able to learn from experiences of other countries (3) |
| **Jordan** | Advice provided on engaging with various knowledge exchange activities via email (1) | None specified (1) |
| **El Salvador** | None specified (1) | Improved international relations and collaborations (1) |
| **Colombia** | Advice provided on engaging with various knowledge exchange activities in other countries (e.g El Salvador and South America) (1) | Increased knowledge sharing on tobacco control issues especially taxation (3) |
| **Egypt** | None specified (0) | - (1) |
| **Myanmar** | Financial and technical support provided to engage in tobacco control activities (2) | Able to access advice and support globally (3) |
| **Samoa** | Financial support provided to attend workshops in Cape Town (1) | Able to learn from experiences of other countries (3) |
| **Georgia** | Financial support, training and workshops for knowledge exchange activities (2) | Learnings and experiences of other countries adopted in Georgian context (3) |
| **Sri Lanka** | Financial support provided to arrange a study tour of enforcement officers- Public Health Inspectors, Police and Custom Officers to Singapore (1) | Sharing experiences and observing enforcement activities were valuable (3) |
| **Cabo Verde** | None specified (0) | - (1) |
| **Chad** | None specified. (0) | - (1) |
| **Nepal** | None specified.(0) | - (1) |
| **Cambodia (taxation only)** |  |  |

| **Table 6c: International and Regional Cooperation—Engagement with International Organisations** | | |
| --- | --- | --- |
| **Country** | **Inputs** | **Progress** |
| **Zambia** | Advice provided to link with CTFK and engage in relevant activities (1) | Increased exposure to the tobacco control organizations in the rest of the world (3) |
| **Sierra Leone** | Advice provided to engage with CTFK and WHO Knowledge Hub (1) | Enhanced regional and international cooperation and collaborations (3) |
| **Jordan** | None specified.(0) | - (1) |
| **El Salvador** | Advice provided to better engage with CTFK and the Union (1) | Enhanced regional and international cooperation and collaborations (3) |
| **Colombia** | Advice provided to engage with the Union (1) | The MoH got involved in writing of several grants for tobacco control research in the country (4) |
| **Egypt** | Advice provided to the MoH to link with WHO Knowledge Hub and the Union (1) | Enhanced regional and international cooperation and collaborations (3) |
| **Myanmar** | None specified (0) | - (1) |
| **Samoa** | Financial support provided to engage with WHO Knowledge Hub and CTFK (1) | More evidence based research (3) |
| **Georgia** | Financial support provided to engage with CTFK and WHO Knowledge Hub (1) | Enhanced regional and international cooperation and collaborations (3) |
| **Sri Lanka** | Advice provided to link with WHO Knowledge Hub (1) | Enhanced regional and international cooperation and collaborations (3) |
| **Cabo Verde** | None specified (0) | - (1) |
| **Chad** | Advice provided to link with WHO Knowledge Hub (1) | The engagement resulted in a tax increase (4) |
| **Nepal** | None specified (0) | - (1) |
| **Cambodia (taxation only)** |  |  |
